# Pharmacological inhibition of the acetyltransferase Tip60 mitigates myocardial infarction injury

**DOI:** 10.1242/dmm.049786

**Published:** 2022-11-07

**Authors:** Xinrui Wang, Tina C. Wan, Katherine R. Kulik, Amelia Lauth, Brian C. Smith, John W. Lough, John A. Auchampach

**Affiliations:** ^1^ Department of Pharmacology and Toxicology, Medical College of Wisconsin, Milwaukee, WI 53226, USA; ^2^ Cardiovascular Center, Medical College of Wisconsin, Milwaukee, WI 53226, USA; ^3^ Department of Cell Biology, Neurobiology and Anatomy, Medical College of Wisconsin, Milwaukee, WI 53226, USA; ^4^ Department of Biochemistry, Medical College of Wisconsin, Milwaukee, WI 53226, USA

**Keywords:** Apoptosis, Cardioprotection, Cardiomyocyte proliferation, Cell-cycle activation, Myocardial infarction, Regeneration, Tip60, TH1834

## Abstract

Pharmacologic strategies that target factors with both pro-apoptotic and anti-proliferative functions in cardiomyocytes (CMs) may be useful for the treatment of ischemic heart disease. One such multifunctional candidate for drug targeting is the acetyltransferase Tip60, which is known to acetylate both histone and non-histone protein targets that have been shown in cancer cells to promote apoptosis and to initiate the DNA damage response, thereby limiting cellular expansion. Using a murine model, we recently published findings demonstrating that CM-specific disruption of the *Kat5* gene encoding Tip60 markedly protects against the damaging effects of myocardial infarction (MI). In the experiments described here, in lieu of genetic targeting, we administered TH1834, an experimental drug designed to specifically inhibit the acetyltransferase domain of Tip60. We report that, similar to the effect of disrupting the *Kat5* gene, daily systemic administration of TH1834 beginning 3 days after induction of MI and continuing for 2 weeks of a 4-week timeline resulted in improved systolic function, reduced apoptosis and scarring, and increased activation of the CM cell cycle, effects accompanied by reduced expression of genes that promote apoptosis and inhibit the cell cycle and reduced levels of CMs exhibiting phosphorylated Atm. These results support the possibility that drugs that inhibit the acetyltransferase activity of Tip60 may be useful agents for the treatment of ischemic heart disease.

## Teaser

**Summary:** Administration of TH1834 mitigated the damaging effects of myocardial infarction in mice, supporting the possibility that Tip60 acetyltransferase inhibitors may be useful for the treatment of ischemic heart disease.

## INTRODUCTION

Death of cardiomyocytes (CMs) after prolonged myocardial ischemia results in loss of muscle cell mass, contractile dysfunction, adverse ventricular remodeling and systolic heart failure. Owing to the onset of proliferative senescence that occurs postnatally, CMs are essentially non-regenerable, preventing regeneration of cardiac muscle. Thus, factors that prevent CMs from proliferating and reduce CM survival constitute compelling targets for the therapeutic amelioration of myocardial infarction (MI). We have pursued the lysine acetyltransferase (KAT) termed Tat-interactive protein, 60 kDa (Tip60; also known as KAT5) as such a multifunctional candidate. Our rationale is based on findings in the cancer biology field showing that Tip60 acetylates multiple non-histone proteins (Li et al., 2019a; Bose et al., 2019; Lee et al., 2013; Rajagopalan et al., 2017; Chen et al., 2019) that regulate these functions. For example, Tip60 activates apoptosis by acetylating p53 (also known as TP53), which in turn trans-activates pro-apoptotic genes (Sykes et al., 2006; Tang et al., 2006). Tip60 also inhibits cell proliferation by acetylating ataxia–telangiectasia mutated (Atm) (Sun et al., 2010; Sun et al., 2009; Sun et al., 2005), thereby initiating the DNA damage response (DDR) culminating in cell-cycle inhibition. We recently reported that Tip60 initiates these activities in CMs at early stages of postnatal heart development (Wang et al., 2021).

To address the role of Tip60 in the ischemic heart, in which it is robustly expressed (McAllister et al., 2002; Fisher et al., 2012), we conditionally disrupted the *Kat5* gene encoding Tip60 in CMs of adult mice 3 days after induction of MI produced by permanent left coronary artery ligation. As we recently reported (Wang et al., 2022), Cre-mediated depletion of Tip60 markedly preserved cardiac function for up to 28 days post-MI and limited the extent of scarring by ∼30%. These findings were accompanied by reduced CM apoptosis, diminished induction of DDR markers in CMs and activation of the CM cell cycle. These findings demonstrated that genetic depletion of Tip60 markedly protected against the damaging effects of MI, which might be explained by enhanced CM proliferation and survival (Wang et al., 2022).

To follow up these findings in a therapeutic context, we are now addressing whether administration of small-molecular-mass (MW) drugs that inhibit Tip60 acetyltransferase activity produces similar beneficial effects. The experiments reported here describe our initial results examining the novel Tip60 inhibitor TH1834, which was recently developed through *in silico* modeling efforts of the Tip60 acetyltransferase domain informed by the antimicrobial medication pentamide. Similar to the effect of disrupting the *Kat5* gene, daily intraperitoneal administration of TH1834 in mice from day 3 until day 16 post-MI resulted in improved systolic function throughout the 28-day experimental timeline. Also similar to hearts with *Kat5* disruption, TH1834-treated hearts removed at day 28 post-MI exhibited reduced apoptosis and scarring, and increased activation of the CM cell cycle. These findings warrant further investigation toward the ultimate goal of utilizing Tip60 inhibitors for the treatment of ischemic heart disease.

## RESULTS

### TH1834 experimental strategy

**Fig. 1.**
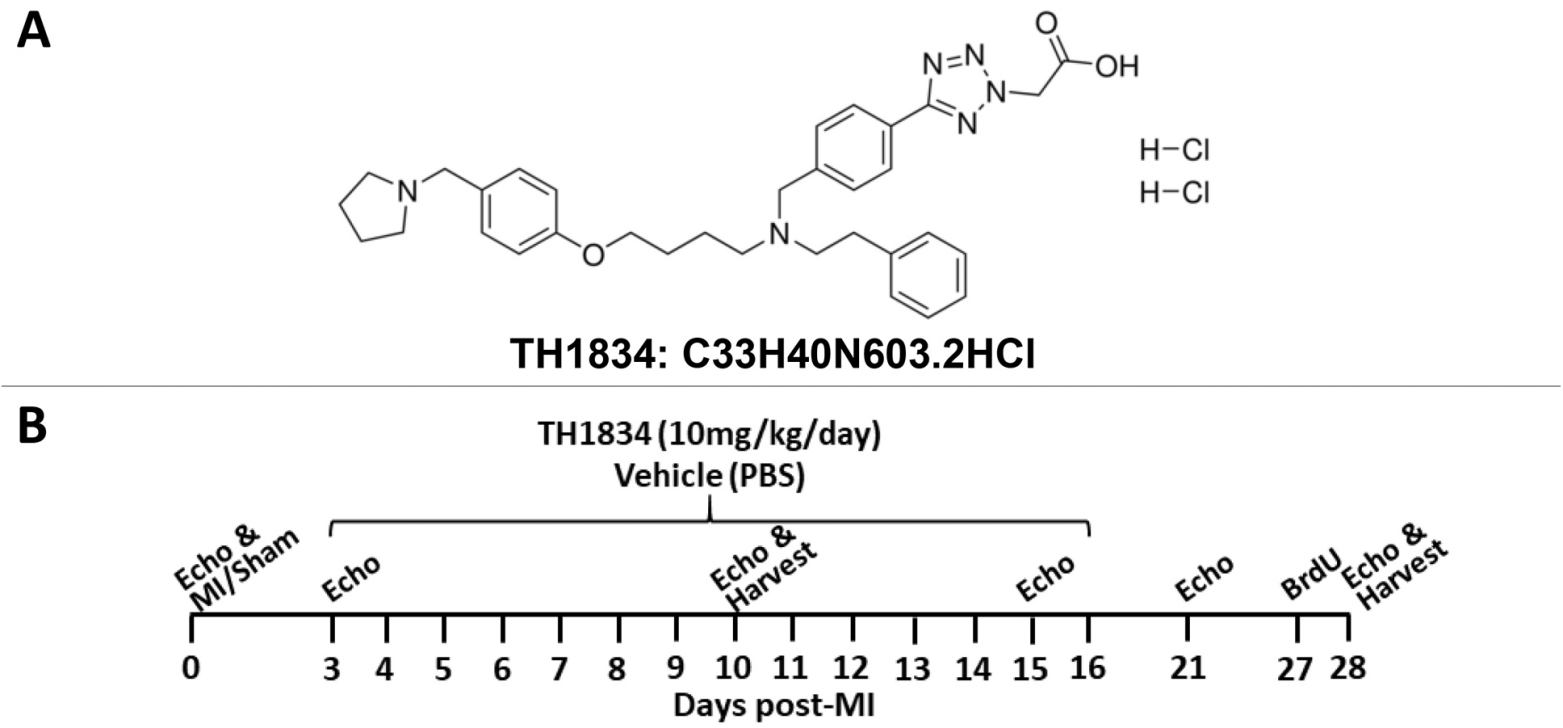
**Experimental strategy.** (A) TH1834 structure. (B) Experimental timeline: hearts of CL57/Bl6 adult mice were infarcted or subjected to sham surgery, followed 3[Supplementary-material sup1]days later by daily intraperitoneal injection of TH1834 (10 mg/kg) for 14 consecutive days. MI, myocardial infarction.

Our objective was to assess whether the damaging effects of MI could be reduced by subsequent pharmacological inhibition of the acetyltransferase domain of Tip60. As shown in Table S1, among commercially developed anti-cancer drugs designed to target KATs, most non-selectively target the similarly structured acetyltransferase domain that is shared by KATs including Tip60 (Brown et al., 2016). Curiously, utilization of anacardic acid, MG149, C646, curcumin and garcinol – agents that non-selectively target KATs – to treat cardiac insult has yielded results that are generally positive (Table S1). To our knowledge, pentamidine, NU9056 and TH1834, which are relatively Tip60 specific, have not been utilized for this purpose. We decided to initially interrogate TH1834, based on its structure (Fig. 1A), which was rationally designed to bind a unique pocket within the acetyltransferase domain of Tip60 (Gao et al., 2014; Brown et al., 2016). As shown in the experimental timeline (Fig. 1B), which is similar to the strategy we previously employed to genetically target *Kat5* (Wang et al., 2022), we assessed the effect of daily intraperitoneal administration of 10 mg/kg TH1834, beginning on day 3 after MI or sham surgery, for 14 consecutive days, ending on day 16 post-MI. During this period, cardiac function was monitored by echocardiography at the indicated intervals (Fig. 1B). On day 10 or 28 post-MI, hearts were removed for histological assessment.

### Treatment with TH1834 improves cardiac function post-MI

**Fig. 2.**
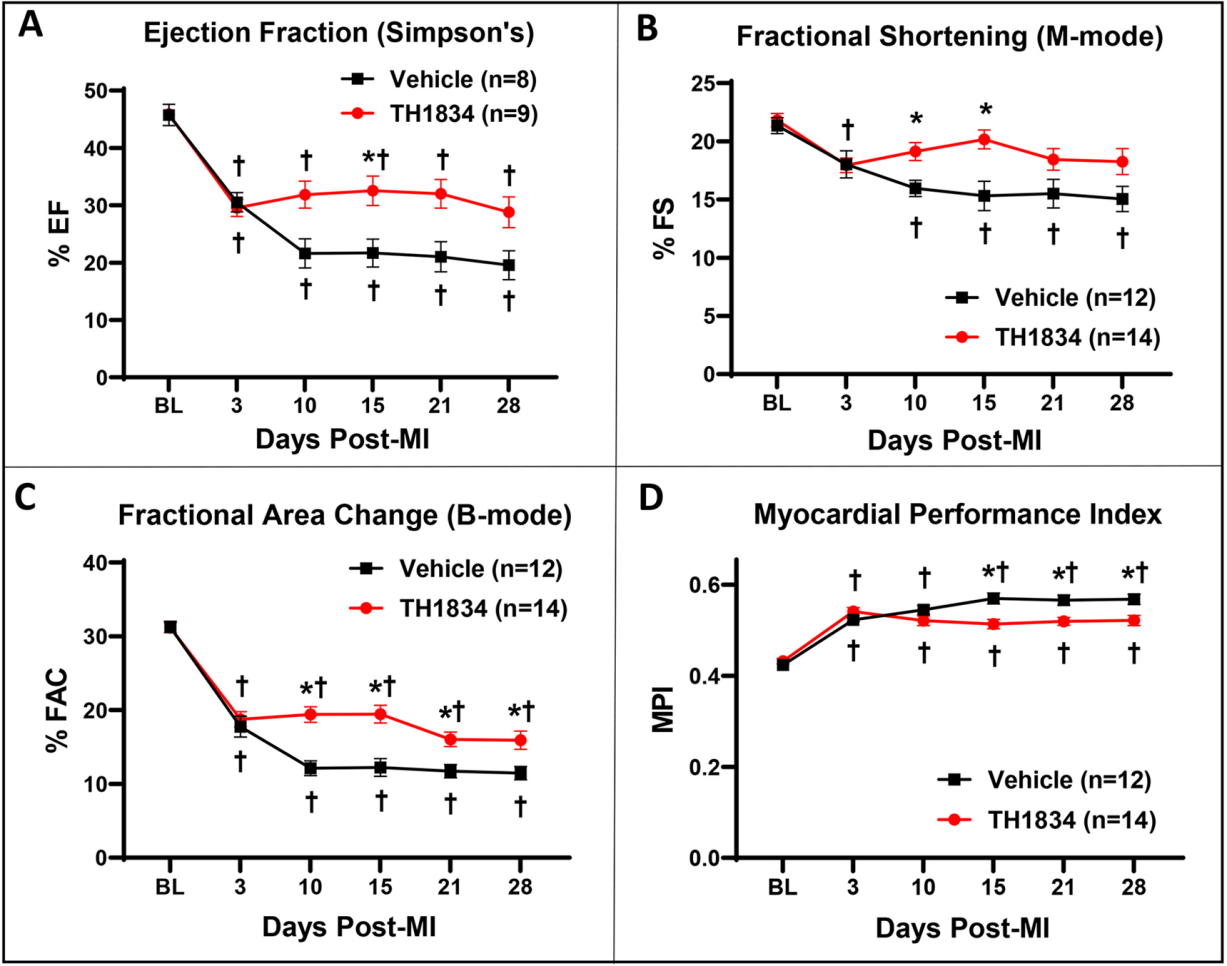
**Administration of TH1834 preserves cardiac function after MI.** Echocardiography was performed at the indicated intervals. (A-D) Indices of left ventricular function: ejection fraction (EF; A), fractional shortening (FS; B), fractional area change (FAC; C) and myocardial performance index (MPI; D). Beginning on day 10, in comparison with vehicle-treated controls, mice treated with TH1834 exhibited improved function. Additional parameters are listed in Tables S2-S5. Echocardiographic data (mean±s.e.m.) were analyzed by two-way repeated measures ANOVA followed by Dunnett's (effect of time) and Bonferroni's (effect of genotype) multiple comparisons. *P<0.05 versus vehicle; ^†^*P*<0.05 versus baseline value on day 0.

The mode of cardiac injury employed was designed to generate uniform infarctions in the inferior half of the ventricle by permanently ligating the left main coronary artery below the tip of the left atrium. Treatment with TH1834 was initiated on day 3 post-MI to avoid potential effects of the drug on initial infarct size and to ascertain effects on post-MI remodeling. As per the timeline (Fig. 1B), echocardiography was performed on infarcted mice at intervals up to 28 days post-MI. As shown in Fig. 2, in comparison with cardiac function at baseline, dysfunction of all echocardiographic indices was observed at all post-MI timepoints in both control and TH1834-treated mice, with the notable exception that fractional shortening (FS) (Fig. 2B; Table S2) in TH1834-treated mice was preserved by 10 days post-MI and thereafter. Ejection fraction (EF) calculated by both the B-mode measurements (Table S3) and Simpson's method (Fig. 2A; Table S4) were comparable, and were significantly improved by administration of TH1834. Moreover, comparison of control and TH1834-treated mice at each timepoint revealed that, beginning on day 10 post-MI, the TH1834 group exhibited improved fractional area change (FAC) (Fig. 2C), myocardial performance index (MPI) (Fig. 2D) and stroke volume (SV) (Tables S3 and S4), effects that were sustained until the experiment was terminated on day 28 post-MI when hearts were removed for histology. As shown in Fig. S1, indices of cardiac function were not altered in TH1834-treated sham-operated mice, indicating that improved function in the MI experiments was not due to a general effect of the drug to enhance cardiac inotropy, but rather must be explained by alternative mechanisms. Details and summaries of echocardiographic data, and heart/body weight ratios, are provided in Tables S2-S5.

### Post-MI treatment with TH1834 reduces scarring

**Fig. 3.**
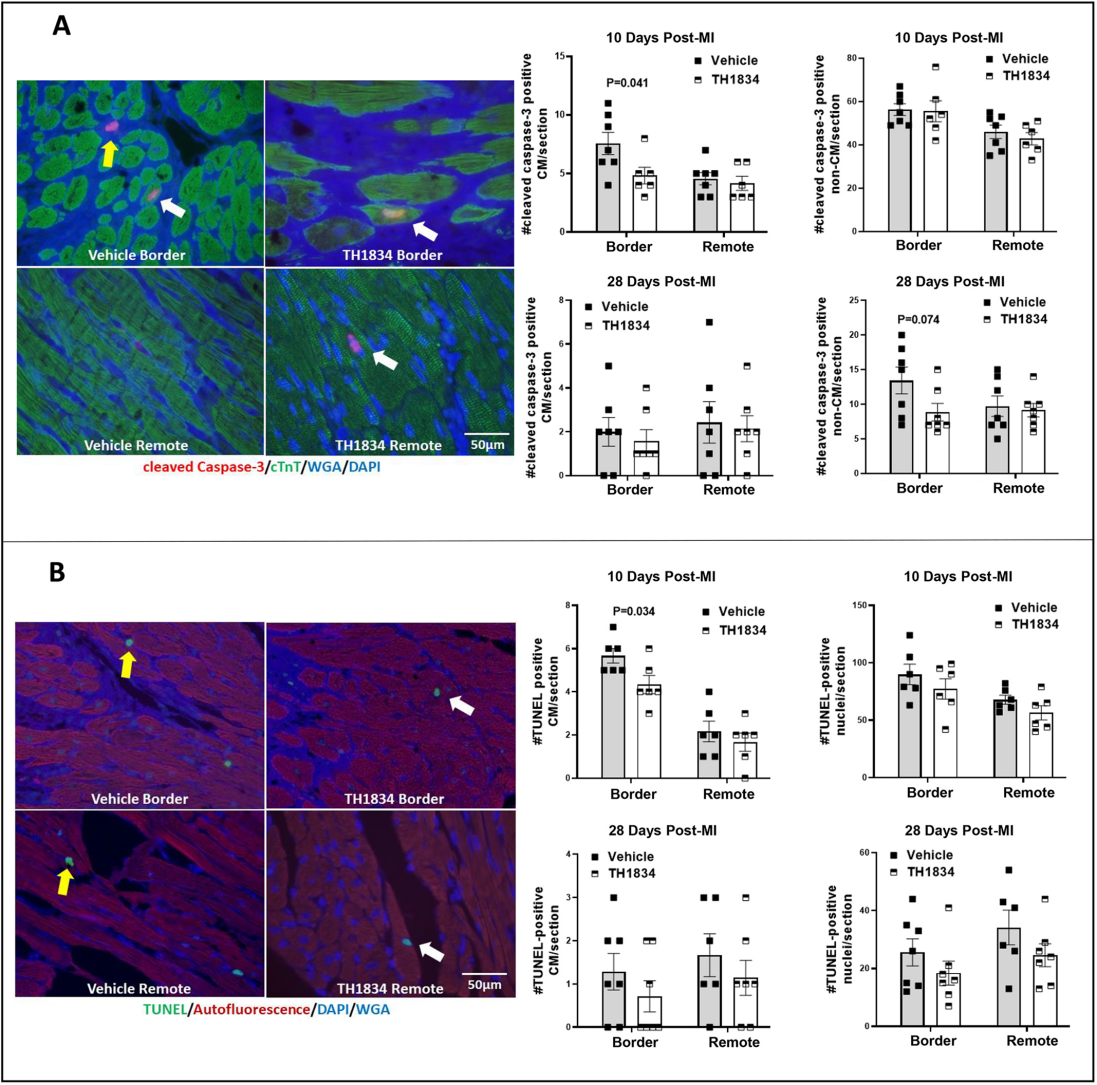
**Effects of TH1834 treatment on apoptosis in infarcted mouse hearts.** (A) Representative immunostains and quantification of apoptosis according to cleaved caspase-3 positivity. (B) Representative immunostains and quantification of apoptosis according to TUNEL positivity. White and yellow arrows indicate cardiomyocytes (CMs) and non-CMs, respectively. Data (mean±s.e.m.) were compared by unpaired, two-tailed Student's *t*-tests with Welch's correction. cTnT, cardiac troponin-T; WGA, wheat germ agglutinin.

Because Tip60 is pro-apoptotic (Tang et al., 2006; Sykes et al., 2006), it was of interest to assess whether treatment with TH1834 on days 3-16 post-MI reduced apoptosis, as well as myocardial scarring due to infarction, in hearts removed for histological inactivation at days 10 and 28 post-MI. Apoptosis was assessed by cleaved caspase-3 (Fig. 3A) staining and by terminal deoxynucleotidyl transferase dUTP nick end labeling (TUNEL) (Fig. 3B). Enumeration of caspase-3-positive CMs, as identified by cardiac troponin-T (cTnT; also known as TNNT2)/wheat germ agglutinin (WGA) staining, revealed significantly reduced numbers in the border zone at day 10 post-MI. Staining of TUNEL-positive nuclei in CMs within the border zone yielded a similar result.

**Fig. 4.**
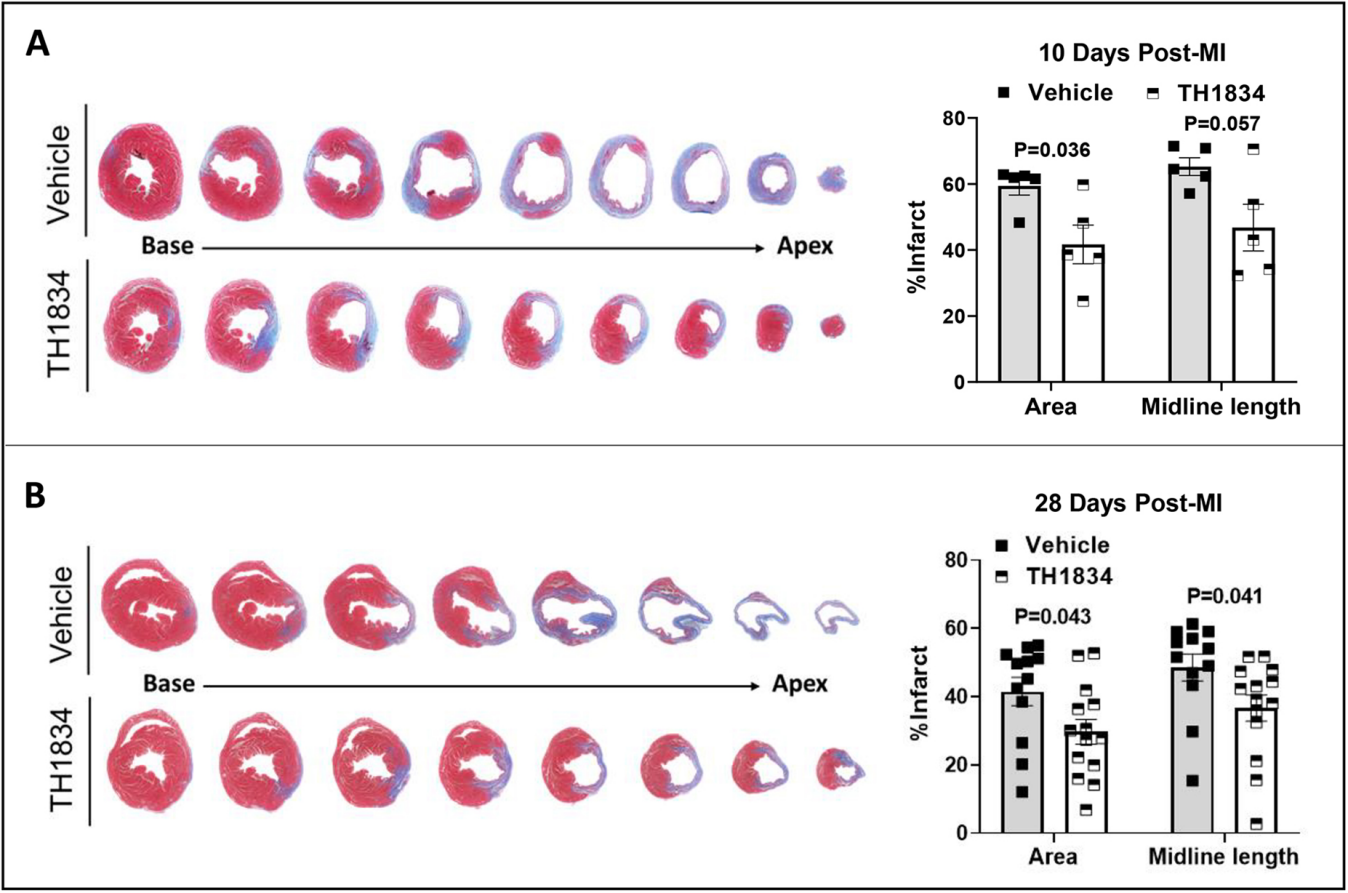
**Effects of TH1834 treatment on scarring in infarcted mouse hearts.** (A,B) Representative trichrome-stained cross-sections (left) and scar size quantified by measuring area and midline length below the ligation site (right) at days 10 (A) and 28 (B) post-MI. Sections were taken at 0.3 mm intervals; blue staining shows the area of the scar. Data (mean±s.e.m.) were compared by unpaired, two-tailed Student's *t*-tests with Welch's correction.

To directly evaluate myocardial damage, the extent of scarring was quantitatively assessed by digitizing blue-stained areas, which are indicative of collagen deposition, in trichrome-stained transverse sections removed at 0.3 mm intervals along the axis between the apex and ligation site (Fig. 4, left). In accordance with improved function seen by echocardiography, hearts in TH1834-treated mice exhibited significantly diminished scarring at 10 and 28 days post-MI, as indicated by ∼25% reductions in both the area and midline length parameters (Fig. 4, right).

### Post-MI treatment with TH1834 activates the cell cycle in CMs

**Fig. 5.**
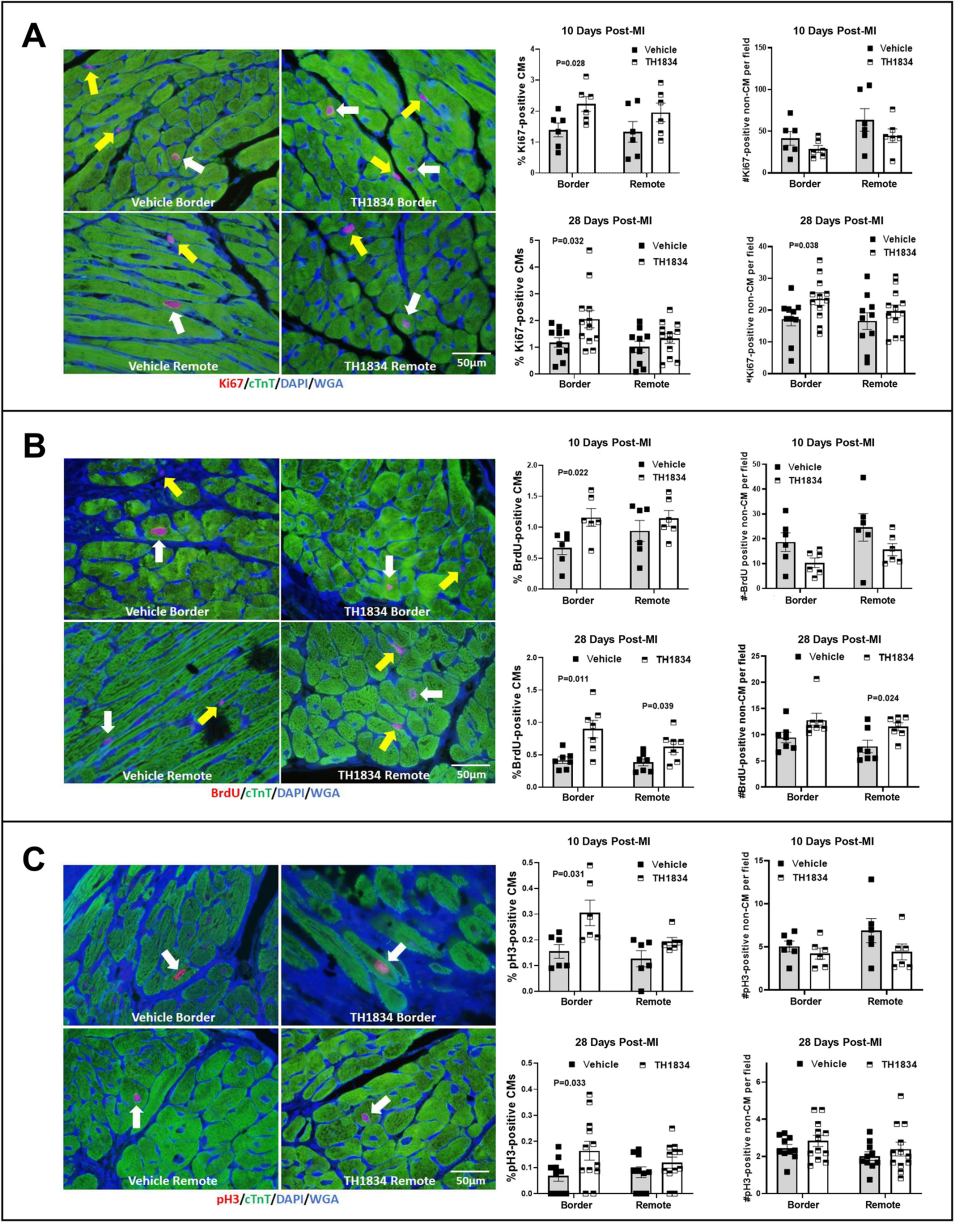
**Increased CM cell-cycle activation in infarcted mice treated with TH1834.** (A-C) Microscopic images in the left column are representative immunostains of each cell-cycle activation marker, as indicated by red nuclear fluorescence. CMs were identified by cytoplasmic expression of cTnT (green fluorescence). White and yellow arrows indicate CMs and non-CMs, respectively. The middle column of each panel shows percentages of CMs expressing Ki67 (A), BrdU (B) and pHH3 (C) at 10 and 28 days post-MI. The right column shows numbers of positive non-CMs, as assessed by evaluating at least 1000 CMs in six 200× fields per zone within each heart. Data (mean±s.e.m.) were compared using unpaired, two-tailed Student's *t*-tests with Welch's correction.

To determine whether treatment of mice with TH1834 beginning 3 days post-MI mimicked the effects of disrupting the *Kat5* gene on cell-cycle activation observed on day 28 post-MI, immunostaining by Ki67 (also known as MKI67) (Fig. 5A), 5′-bromo-2′-deoxyuridine (BrdU) (Fig. 5B) and phosphohistone H3 (pHH3) (Fig. 5C) was assessed. These markers identify cells activated throughout the entire cell cycle, during S-phase only, and in early M-phase, respectively. CMs were distinguished from non-CMs by cytoplasmic co-staining with cTnT. As shown in the middle column of each panel of Fig. 5, the percentage of CMs exhibiting each of these cell-cycle activation markers was significantly increased in CMs within the border zone of infarcted hearts of mice that had been treated with TH1834, but not with vehicle, at both 10 and 28 days post-MI; trends toward increases in the remote zone were also observed. Similar trends toward cell-cycle activation were observed in non-CMs at day 28 post-MI, i.e. hearts in the TH1834-treated group showed more Ki67- and BrdU-positive non-CMs in the border and remote zones, respectively (right column in Fig. 5A,B); although this phenomenon, which was also observed in infarcted hearts following disruption of the *Kat5* gene, is unexplained, we previously speculated that it represents a CM-based paracrine effect and/or compensated changes resultant from MI (Wang et al., 2022).

**Fig. 6.**
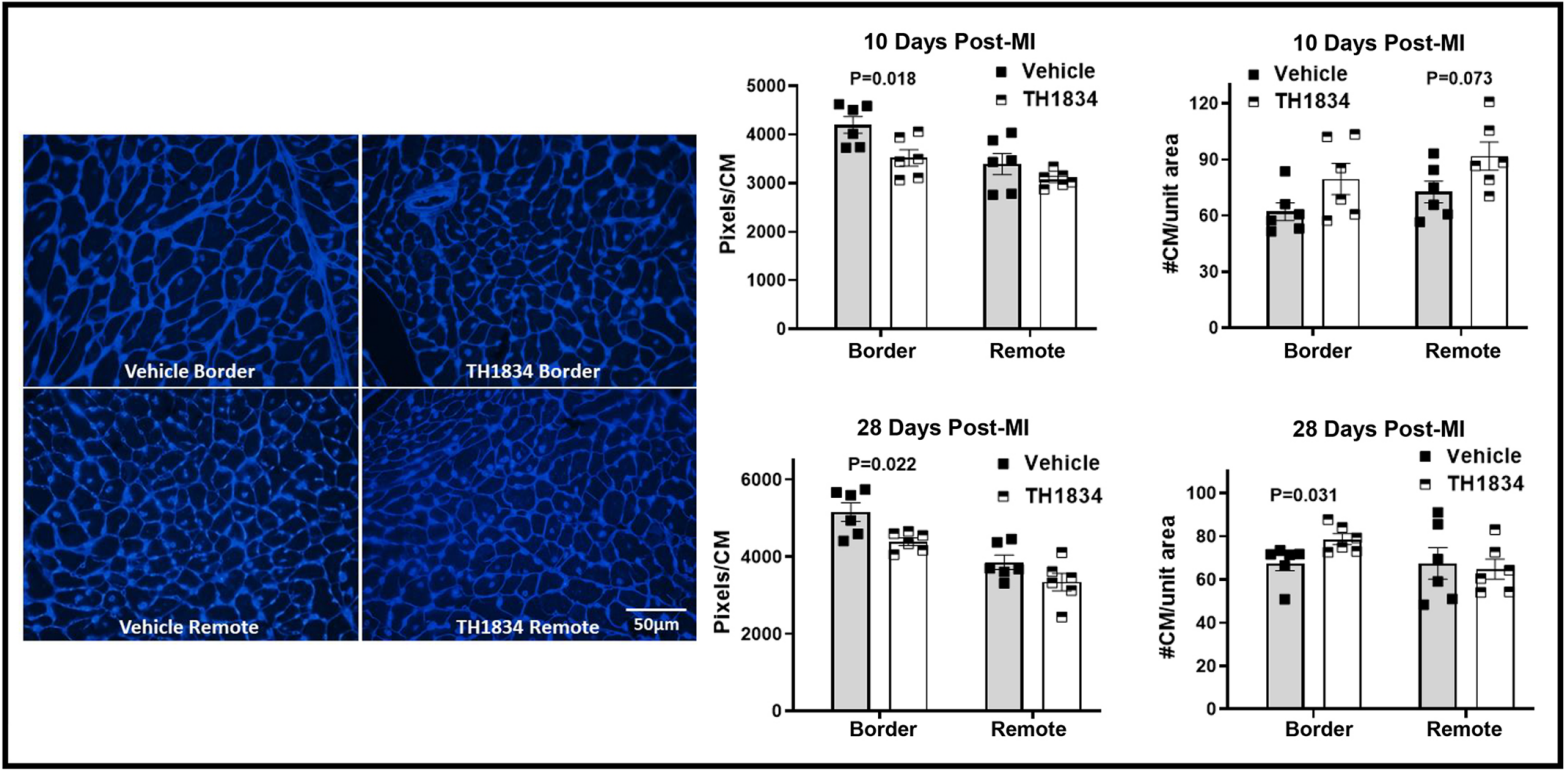
**Decreased size and increased density of CMs in the border zone of hearts treated with TH1834.** Left column: representative areas in the border zone containing transversely sectioned CMs stained with WGA. Middle column: bar graphs indicating reduced size (pixels/CM) of CMs in the border zone at 10 and 28 days post-MI. Right column: bar graphs indicating increased density (number of CMs/unit area) of CMs. Counts were made by ImageJ analysis of >300 transversely sectioned CMs per zone in each heart. Data (mean±s.e.m.) were compared using unpaired, two-tailed Student's *t*-tests with Welch's correction.

To assess whether CM hypertrophy may have contributed to improved cardiac function in TH1834-treated mice, CM size was estimated by quantitating pixels in transversely sectioned CMs circumscribed by WGA fluorescent staining (Fig. 6). This indicated that CM size was not increased in hearts of TH1834-treated mice. In fact, CMs in hearts of mice that had been treated with TH1834 for 14 consecutive days were smaller than those in hearts of vehicle-treated mice (Fig. 6, middle column), suggesting that bona fide CM proliferation was induced by inactivating Tip60, a possibility consistent with increased density of CMs in TH1834-treated hearts (Fig. 6, right column).

Also, because de-differentiation of CMs precedes cytokinesis and proliferation (Zhu et al., 2021), we evaluated gap junction integrity, as indicative of differentiated status, by immunostaining connexin-43 in hearts of TH1834-treated mice (Fig. S2). Similar to the gap junction dysmorphology we recently described in Tip60-depleted hearts (Wang et al., 2022), this indicated that the pattern of connexin-43 staining was disrupted in hearts of the majority of mice treated with TH1834.

### Post-MI treatment with TH1834 reduces expression of Tip60-regulated genes and numbers of CMs positive for the DDR marker phosphorylated Atm (pAtm)

**Fig. 7.**
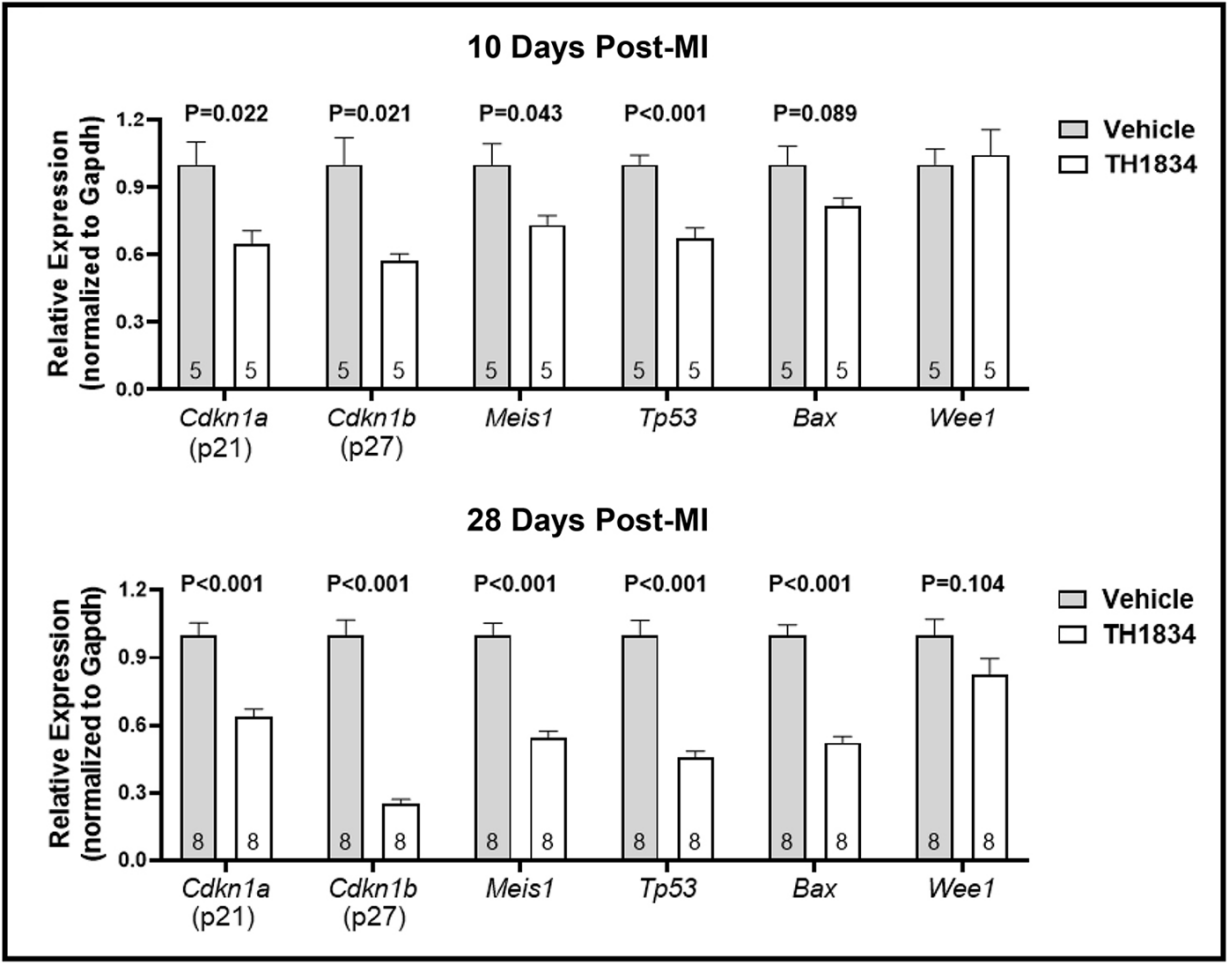
**Reduced expression of molecular targets of Tip60 in TH1834-treated hearts.** Heart tissue removed at 10 (top) and 28 (bottom) days post-MI was subjected to quantitative RT-PCR determinations of genes that we previously determined to be reduced following genetic depletion of Tip60 (Wang et al., 2022). Data are presented relative to *Gapdh*; similar results were obtained when normalized to an alternative housekeeping gene (*Rpl37a*). Each biological replicate (group size indicated within each bar) is the average of three technical replicates. Data (mean±s.e.m.) were compared using unpaired, two-tailed Student's *t*-tests with Welch's correction.

**Fig. 8.**
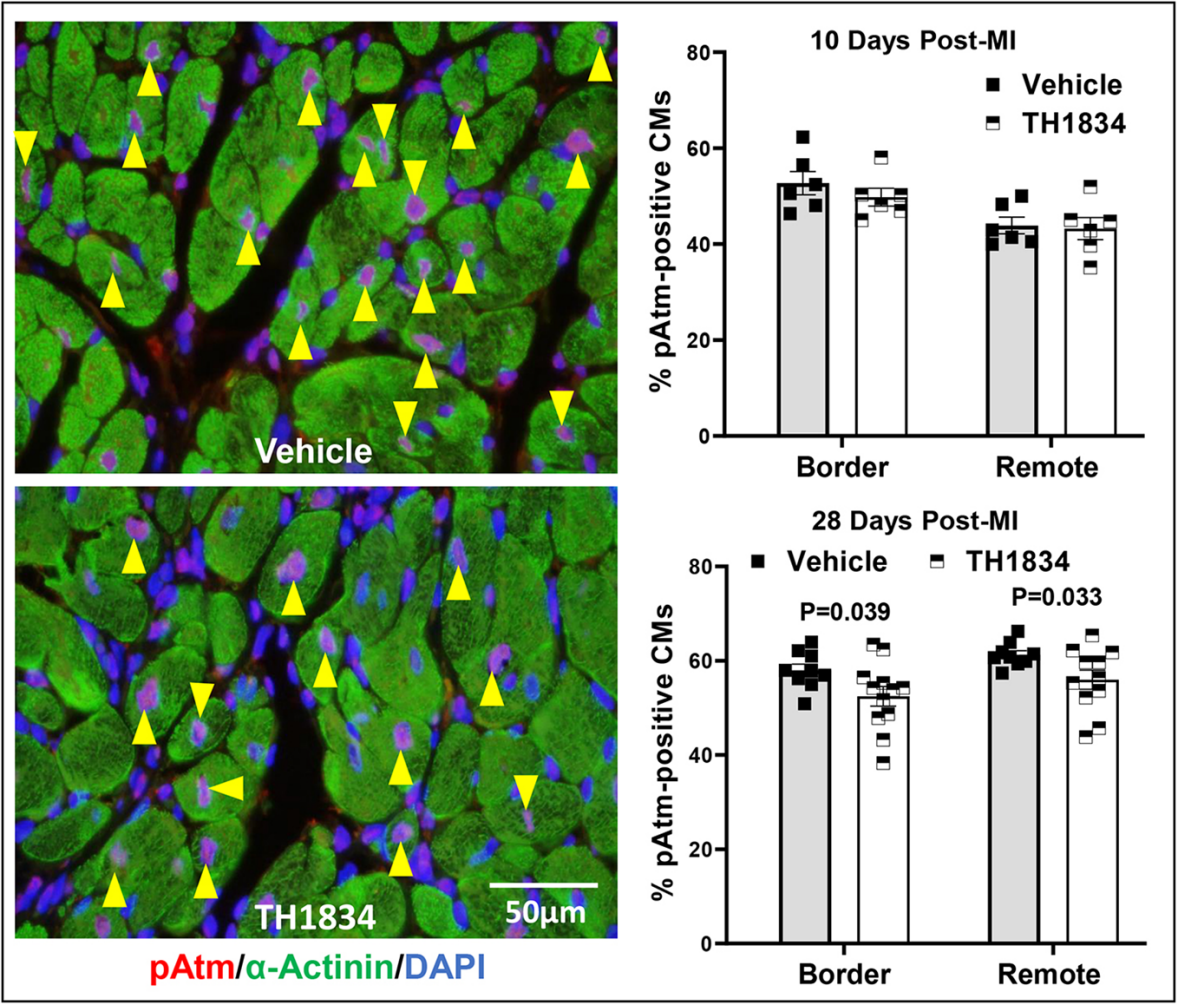
**Reduced numbers of phosphorylated Atm (pAtm)-positive CMs in TH1834-treated hearts.** Left column: representative pAtm/α-actinin double-stained images; yellow arrowheads indicate pAtm-positive nuclei identified according to the ground rules described in the Materials and Methods. Right column: graphs depicting the percentage of pAtm-positive CMs, based on evaluating a minimum of 1000 CMs in the border and remote zones. Data (mean±s.e.m.) were compared using unpaired, two-tailed Student's *t*-tests with Welch's correction.

To confirm specificity of TH1834, expression of six genes [*Cdkn1a* (p21), *Cdkn1b* (p27), *Meis1*, *Tp53* (p53), *Bax*, and *Wee1*] shown in our previous studies (Wang et al., 2021, 2022) to be downregulated in the heart after genetic depletion of Tip60 from CMs was assessed by quantitative RT-PCR (qPCR). In addition, numbers of CMs positive for the DDR marker pAtm, which were also found to be reduced in our previous genetic depletion studies, were quantified by immunostaining (Wang et al., 2021, 2022). Except for *Wee1*, all Tip60-regulated genes were reduced in TH1834-treated hearts (Fig. 7), as were numbers of pAtm-positive CMs in both the border and remote zones at the 28-day timepoint (Fig. 8). Collectively, these findings provide strong evidence that administration of TH1834 effectively inhibited Tip60 activity.

## DISCUSSION

The goal of this study was to test the hypothesis that, following MI, pharmaceutical inhibition of Tip60, a KAT known to promote apoptosis and inhibit the cell cycle in cultured cells, protects and regenerates the *in vivo* myocardium by limiting apoptosis and permitting re-activation of the CM cell cycle, respectively. The findings described above are consistent with the possibility that post-MI treatment with a systemically delivered anti-Tip60 drug can diminish scarring while activating the CM cell cycle, resulting in improved cardiac function.

As recently reviewed, accumulating evidence indicates that the myocardial acetylome is disrupted in various cardiovascular diseases (Li et al., 2020c). Although lysine deacetylases (KDACs) and KATs have therefore emerged as therapeutic targets, KATs are receiving less attention (Li et al., 2020c). As shown in Table S1, several drugs are available that inhibit the conserved acetyltransferase domain shared by the ∼20 members (Voss and Thomas, 2018) of the KAT family. Among these, anacardic acid (Peng et al., 2015; Li et al., 2019b; Mao et al., 2021), MG149 (Yu et al., 2018), C646 (Rai et al., 2019; Su et al., 2020), garcinol (Balasubramanyam et al., 2004; Li et al., 2020b) and curcumin (Morimoto et al., 2008; González-Salazar et al., 2011; Yang et al., 2013; Sunagawa et al., 2014; Xiao et al., 2016; Sunagawa et al., 2018; Li et al., 2020a) – all of which non-selectively target acetyltransferase domains in KATs – have been reported to limit dysfunction caused by cardiac ischemia and hypertrophy. Among these, the polyphenol curcumin, which inhibits p300 [Kat3b; also known as EP300; half-maximal inhibitory concentration (IC_50_) ∼40 µM] with reasonable selectivity (Wu et al., 2009) in comparison with Tip60 (IC_50_ ∼200 µM), has received most attention, resulting in improved cardiac function in preclinical and clinical studies (reviewed in Li et al., 2020a). MG149, which inhibits Tip60 and its closely related family member Mof (also known as KAT8) at an IC_50_ of 74 µM and 47 µM, respectively, was recently shown to prevent ischemia–reperfusion injury in mice (Yu et al., 2018). Considered along with our findings utilizing TH1834, these findings justify expanded efforts to treat cardiovascular disease by targeting individual members of the KAT family with improved specificity.

To address the specificity of TH1834 for known targets of Tip60, qPCR was used to monitor the expression of genes that we previously interrogated to assess the effects of genetically depleting Tip60 in CMs of neonatal and adult hearts (Wang et al., 2021, 2022). As shown in Fig. 7, with the exception of *Wee1* (which nonetheless trended downward on day 28), expression of all of these genes was significantly reduced in hearts of TH1834-treated mice. Reduced expression of the gene encoding the cell-cycle inhibitor p21, *Cdkn1a*, which was unchanged in our previous studies, is of particular interest because p21 has been shown to be a direct target of Tip60 (Lee et al., 2013; Li et al., 2019a). Reduced expression of *Cdkn1a* is consistent with reduction of *Tp53*, which encodes p53, because p53 transactivates the *Cdkn1a* promoter (Legube et al., 2004); interestingly, p53 is also a direct target of Tip60 (Sykes et al., 2006; Tang et al., 2006). Reduced expression of *Cdkn1a* is also consistent with that of *Meis1*, because Meis1 protein is required to activate *Cdkn1a* transcription (Mahmoud et al., 2013). Moreover, the strong downregulation shown in Fig. 7 of *Cdkn1b* (p27), a member of the same family of cyclin-dependent kinase (CDK) inhibitors, is consistent with our observations of reduced *Cdkn1b* expression and p27 protein in neonatal and adult hearts in which Tip60 is genetically depleted (Wang et al., 2021, 2022); we are currently assessing whether p27 is also a direct target of Tip60. Also, the reduced expression of *Tp53* and *Bax* in TH1834-treated hearts is consistent with the well-accepted findings that Tip60-mediated acetylation of p53 promotes apoptosis via activation of the *Bax* promoter, which is also consistent with results in Fig. 3, showing that apoptosis is reduced in hearts of TH1834-treated mice. Finally, because autophosphorylation of Atm and initiation of the DDR requires prior acetylation of Atm by Tip60 (Sun et al., 2010; Sun et al., 2009; Sun et al., 2005), the immunostaining results in Fig. 8 showing that numbers of pAtm-positive CMs were reduced in TH1834-treated hearts at the 28-day timepoint provide further confirmation of the specificity of TH1834 for Tip60.

The mode (intraperitoneal), dosage (10 mg/kg/day) and duration (14 days) selected for deploying TH1834 in this study were estimated based on previous results using the KAT inhibitors listed in Table S1 and above. In ongoing studies, we are addressing the response of infarcted hearts to various dosages and durations of treatment with TH1834, with emphasis on its effects at earlier timepoints and abbreviated durations of treatment. Optimal regimens must ultimately be assessed for efficacy in animal models that mimic common co-morbidities (atherosclerosis, metabolic disease) associated with ischemic heart disease and in the context of aging. Also, although we have observed no untoward effects of TH1834 (Fig. S1), the long-term effects, if any, of transiently inhibiting Tip60, which is considered to possess tumor suppressor function, are being carefully monitored. Studies are also underway to assess whether activation of the CM cell cycle shown in Fig. 5 heralds bona fide proliferation and expansion of CM numbers, and whether treatment with TH1834 may modulate the post-MI inflammatory response that contributes to infarct expansion and adverse remodeling. This later point is particularly intriguing considering recent work showing that Tip60, via acetylation of FOX3P (Xiao et al., 2014), controls numbers and activity of regulatory T cells, a class of immunosuppressive lymphocytes that have been implicated in post-MI remodeling by both suppressing inflammation and stimulating CM proliferation (Zacchigna et al., 2018). Finally, alternative to targeting the acetyltransferase domain of Tip60, in order to improve specificity, we are designing agents to target its unique chromodomain, which has been shown to be necessary for acetyltransferase function (Sun et al., 2009).

In summary, the findings reported here show that TH1834 administration on days 3-16 post-MI mimics the functional benefits of genetically depleting Tip60, which is accompanied by diminished scarring, enhanced CM cell-cycle activation, reduced CM apoptosis and increased CM density, effects that are in accordance with reduced expression of genes that promote apoptosis and inhibit the cell cycle. These findings support the translational potential of specific inhibitors of Tip60 for limiting the damaging effects of myocardial ischemic injury.

## MATERIALS AND METHODS

### Animal care and experimentation

These experiments adhered to the National Institutes of Health (NIH) Guide for the Care and Use of Laboratory Animals (NIH Pub. Nos. 85-23, Revised 1996). All protocols described in the authors' Animal Use Application (AUA #000225), which were approved by the Medical College of Wisconsin's Institutional Animal Care and Use Committee (IACUC), were adhered to in this study. The IACUC has Animal Welfare Assurance status from the Office of Laboratory Animal Welfare (A3102-01). For these experiments, C57Bl/6 mice were purchased from The Jackson Laboratory.

### Echocardiography

Echocardiography was performed prior to induction of MI or sham surgery and at subsequent intervals using a VisualSonics 3100 high-frequency ultrasound imaging system. This was performed on mice lightly anesthetized with isoflurane delivered via a nose cone (1.0-1.5%). Parasternal long-axis, short-axis and apical four-chamber views were obtained using a transducer (MX550D) operating at 30-40 mHz. Short-axis views in M-mode assessed left ventricular anteroposterior internal diameter (LVID), anterior wall thickness (LVAW) and posterior wall thickness (LVPW) at end-diastole (d) and end-systole (s) at the mid-ventricular level. Long-axis views in B-mode assessed left ventricular internal area (LVA) and length (L) at end-diastole and end-systole. Three short-axis views (evenly spaced around a mid-level view at the papillary muscles, one towards the apex or distal portion of the heart and one towards the base or proximal portion of the heart) assessed internal area (AreaMid, AreaDist and AreaProx) at end-diastole and end-systole. Left ventricular systolic function was assessed by the following: (1) % FS, calculated as [(LVIDd−LVIDs)/LVIDd]×100; (2) % FAC, calculated as [(LVAd−LVAs)/LVA d]×100; and (3) % EF, calculated as (end-diastolic volume−end-systolic volume)/end-diastolic volume, where volumes were estimated by B-mode: 4π/3×L/2×[LVA÷π(L/2)] or (AreaMid+AreaDist+AreaProx)×L/3 (Simpson's method). In addition, global left ventricular function was assessed by calculating the myocardial performance index (MPI) as (isovolumic contraction time+isovolumic relaxation time)/ejection time (Broberg et al., 2003; Tei et al., 1995; Poulsen et al., 2000; Schaefer et al., 2005). Time intervals were obtained from pulsed Doppler waveforms of mitral valve inflow and aortic valve outflow obtained from apical four-chamber views.

### MI

MI was induced, or sham surgery performed, in 10- to 12-week-old male mice under general anesthesia with isoflurane (1.5-2.0%) and mechanical respiration (model 845, Harvard Apparatus) via an endotracheal tube with room air supplemented with 100% O_2_. Electrocardiograms (ECGs; limb lead II configuration) were continuously recorded (Powerlab) via needle electrodes. Rectal temperature was maintained at 37°C using a servo-controlled heating pad. Following anesthesia onset, mice were subcutaneously injected with sustained release meloxicam (4 mg/kg) to manage postoperative pain. A left-sternal thoracotomy was performed to expose the heart, and the pericardium was opened. To target the MI to the lower half of the ventricle, an 8-0 nylon suture was threaded beneath the left main coronary artery at a level below the tip of the left atrium, with the aid of a microscope. Ischemia was induced by tying a permanent suture with a double knot. Coronary occlusion was verified by visually observing blanching of the myocardium distal to the ligature, and by elevation of the ST segment on the ECG. For sham experiments, the sutures were placed as described above but they were not tightened to occlude the artery. The chest wall was closed using polypropylene suture. Recovery was monitored until mice became fully ambulatory.

### Administration of TH1834

Administration of TH1834 was commenced beginning on day 3 post-MI/sham surgery via daily intraperitoneal injections of vehicle (1× PBS) to control mice or PBS containing the Tip60 inhibitor TH1834 (10 mg/kg; C33H40N603.2HCl; MW 641.63) to experimental mice; TH1834 was purchased from Axon Medchem (The Netherlands; cat. #2339). Daily injections were performed for 14 consecutive days, i.e. from days 3-16 post-surgery. On the day before harvest, mice were intraperitoneally injected with BrdU (1 mg). On the day of harvest (10 or 28 days post-MI), mice were euthanized with CO_2_, and hearts were removed and processed for immunostaining (tissue between the apex and ∼1 mm above the suture) or qPCR as described below.

### TUNEL

TUNEL staining was assessed using a DeadEnd Fluorometric TUNEL System (Promega, #G3250) as per the manufacturer's instructions. The total number of TUNEL-positive cells within sections representing the border and remote zones was manually counted while scanning at 400× magnification. TUNEL signal was counted only if confined within a 4′,6-diamidino-2-phenylindole (DAPI)-positive nucleus, and nuclei were scored as TUNEL-positive only if at least 50% of the nucleus contained fluorescent signal. Attempts to immunofluorescently co-stain TUNEL-stained sections for markers of CM identity were unsuccessful, presumably due to removal of antigen during proteinase-K digestion. We therefore identified CMs based on cell size (WGA staining, see below) and autofluorescence signal. Apoptosis was also determined by immunostaining cleaved caspase-3 as described below.

### Myocardial scarring

Myocardial scarring was assessed in Masson trichrome-stained transverse sections (4 µm thick) of hearts removed at 0.3 mm intervals along the axis between the apex and ∼1 mm above the ligation site. Trichrome-stained sections were photographed at 10× magnification on a Nikon SMZ800 microscope, and MIQuant software was used to quantitate infarct size, as previously described (Leach et al., 2017). Results are expressed as the average percentage of area, and the midline length, around the left ventricle.

### WGA staining

WGA staining was performed using Thermo Fisher Scientific #W11263 Alexa Fluor 350 conjugate. Sections mounted on microscope slides were stained with 50 µg/ml WGA in PBS for 10 min at room temperature, followed by thorough washing. Images of CMs in transverse orientation were photographed at 400× magnification and processed using ImageJ software to determine numbers of CMs, and numbers of pixels per CM, as indicative of CM density and CM size, respectively. Briefly, the DAPI (350) channel displaying CMs outlined in cross-section was isolated, followed by thresholding to fill in spaces occupied by CM cytoplasm, then adjusting settings to acquire particle sizes in the 600-infinity range having a circularity of 0.25-1. After results (which were set to ‘include holes’) were obtained, particles representing CMs that were non-transversely sectioned, or blood vessels, were removed.

### Immunostaining and cell counting

On the day before harvest, mice were injected with 1 mg BrdU (Sigma-Aldrich, #B9285). Following removal, hearts were perfused with ice-cold cardioplegic solution and atria were removed. Ventricles were fixed overnight in fresh ice-cold 4% paraformaldehyde/PBS, processed through an ethanol series and embedded in paraffin. Sections (4 µm) mounted on microscope slides were de-waxed, subjected to antigen retrieval (100°C in 10 mM trisodium citrate pH 6.0/0.05% Tween-20 for 20 min) followed by 30 min cooling at room temperature, and blocked with 2% goat serum/0.1% Triton X-100 in PBS. Primary antibodies were diluted in blocking buffer and applied overnight at 4°C; secondary antibodies were applied for 1 h in the dark. Combinations and dilutions of primary and secondary antibodies used to immunostain each target antigen are listed in Table S6.

Microscopy was performed on a Nikon Eclipse 50i microscope equipped with a Nikon DSU3 digital camera. During counting, at least 1000 CMs were evaluated in five to six random 200× photomicrographic fields. CM identity was verified by co-immunostaining cytoplasm with cTnT or α-actinin, and cellular outline with WGA staining. CMs in the border and remote (the area ∼2 mm distal to the infarct boundary) zones relative to the infarction were separately counted. To identify nuclei as pAtm positive, only those at least half-filled with pAtm fluorescence were counted; pAtm-positive nuclei were also confirmed to be DAPI positive.

### qPCR

Heart tissue, previously disrupted by homogenization with a motorized (Kimble 749,540-0000) Teflon pestle and stored at −80°C in TRIzol reagent, was thawed. RNA was immediately purified using PureLink RNA Mini-Kits (Thermo Fisher Scientific, #12183018A), including a genomic DNA removal step (PureLink DNase kit for on-column protocol, Thermo Fisher Scientific, #12185-010), according to the manufacturer's instructions. RNA yield and quality were determined via 260/280 ratio using an Eppendorf Biophotometer Plus instrument.

For synthesis of cDNA, precisely 1 µg of RNA was suspended in 14 µl nuclease-free distilled water (NFDW) to which 4.0 µl 5× VILO reaction mixture (Thermo Fisher Scientific, #11754050) was added. To start the reverse transcription reaction, 2.0 µl 10× SuperScript Enzyme Mix was added, followed by transfer to an Applied Biosystems Veriti 96-well Thermocycler programmed as follows: 10 min at 25°C→60 min at 42°C→5 min at 85°C. cDNA templates were diluted with NFDW to a concentration of 3.125 ng/µl and stored at −20°C.

qPCR was carried out by subjecting each biological replicate (i.e. sample from each individual heart) to triplicate determinations. Each reaction was performed in a total volume of 20 µl in 96-well arrays, each well containing 1× Taqman Fast-Advanced Master Mix (Thermo Fisher Scientific, #4444557), 1× Taqman Probe Kit (Table S7) and 12.5 ng cDNA template. The arrayed samples were amplified in a Bio-Rad CFX96 Real Time System (C1000 Touch) programmed as follows: 2 min at 50°C→20 s at 95°C→3 s at 95°C→30 s at 60°C; the last two steps were repeated 39 times. Results were processed using Bio-Rad CFX Manager 3.1 software.

### Statistics

All determinations were performed in blinded fashion and are reported as means±s.e.m. Echocardiography data were analyzed by a two-way repeated measures ANOVA (time and genotype) to determine whether there was a main effect of time or genotype, or a time–genotype interaction. If global tests showed an effect, post hoc contrasts between baseline and subsequent timepoints within experimental groups were compared by a Dunnett's multiple comparison *t*-test; differences between genotypes at each timepoint were compared by a Student's *t*-test with the Bonferroni correction for multiple comparisons. All other data were compared by an unpaired, two-tailed Student's *t*-test with Welch's correction. *P*<0.05 was considered statistically significant.

## Supplementary Material

10.1242/dmm.049786_sup1 Supplementary information
