# Supplementary material for: Pharmacological inhibition of the acetyltransferase Tip60 mitigates myocardial infarction injury
Source: Dis Model Mech. 2022 Nov 7;16(5):dmm049786. doi: 10.1242/dmm.049786 (PMC9672930; doi:10.1242/dmm.049786)
Supplement: Supplementary information [file dmm-16-049786-s1.pdf]

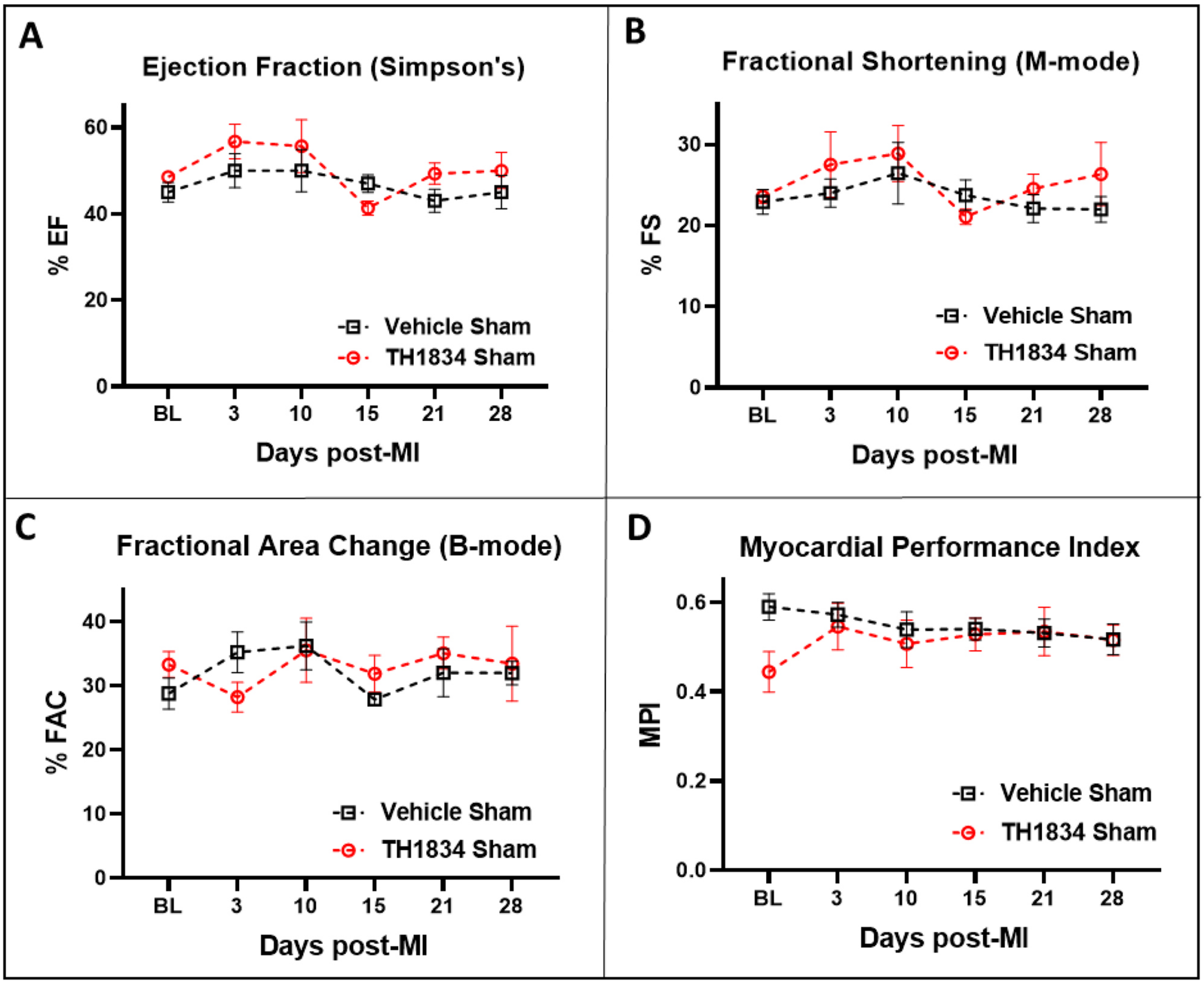

Fig. S1. **No effect of TH1834 on cardiac function of sham-operated mice.** Echocardiography was performed at the indicated intervals up to 28 days after sham surgery. **Panels A-D** show indices of LV function, respectively Ejection Fraction (EF), Fractional Shortening (FS), Fractional Area Change (FAC), and Myocardial Performance Index (MPI). Echocardiographic data (mean ± SEM) were analyzed by two-way repeated measures ANOVA followed by Dunnett's (effect of time) and Bonferroni's (effect of genotype) multiple comparisons. No significant differences in any of the indices of heart function were identified between the two groups.

Disease Models & Mechanisms • Supplementary information

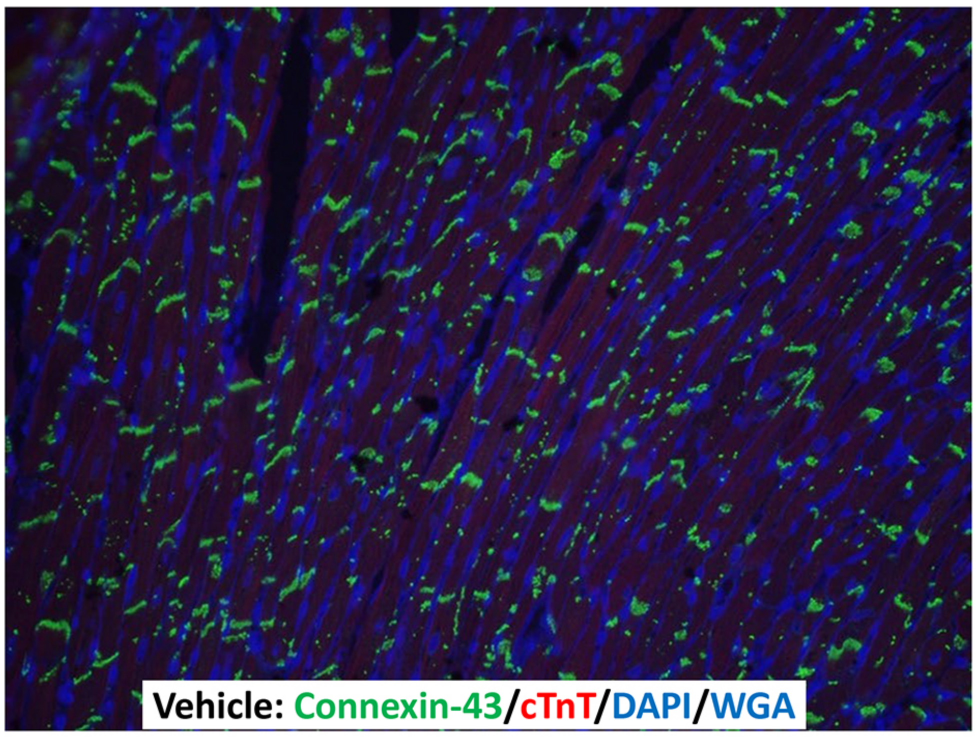

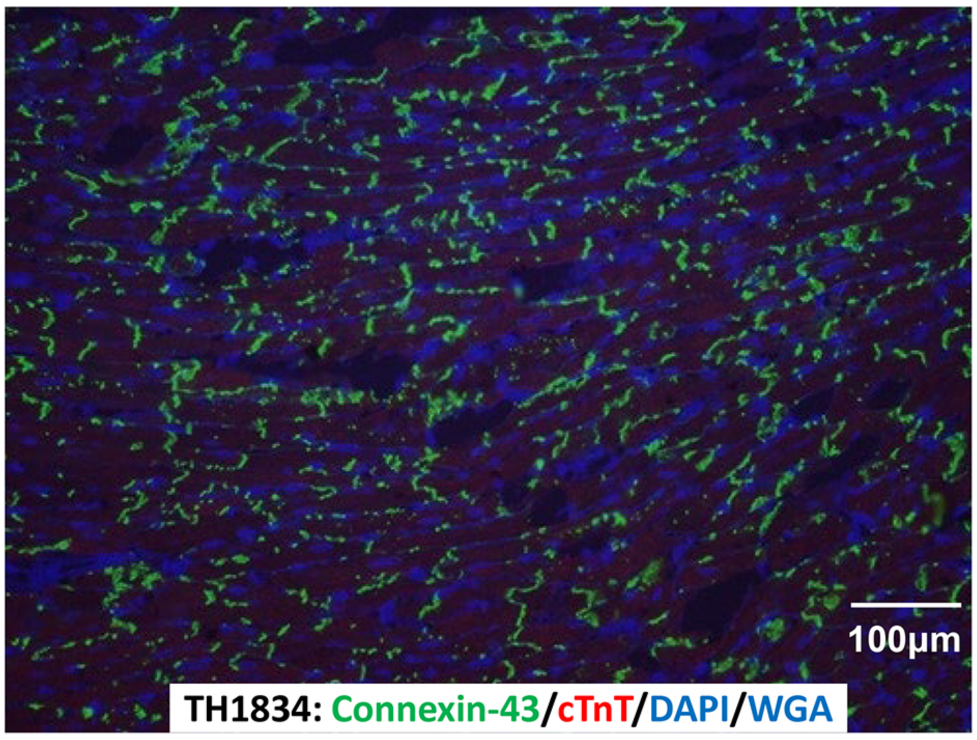

Fig. S2. Connexin-43 dysmorphology in mice treated with TH1834. Representative images photographed at 200x magnification showing immunostaining of Connexin-43 in hearts of Vehicle and TH1834 treated mice at 28 days post-MI, indicating that administration of TH1834 after MI is associated with disruption of the intercalated disk. Although not able to be quantified, this phenotype in TH1834-treated hearts was detected in the majority of TH1834-treated hearts by a blinded observer.

Table S1. Anti-Lysine Acetyltransferase Drugs for Cardiovascular Disease

Drug	Target (IC50; Ki)	Remarks
**Anacardic Acid (AA)**	Tip60 (Kat5; 348 μM[1])<br>PCAF (667 μM[1])<br>p300 (Kat3B; >1000 μM[1])	**Citations:** *Biochimie* 113:1,2015; *J Cell Mol Med* 23:2744,2019; *Cardiovascular Research* 112:555,2016; *Mol Biosyst* 13:714,2017; *Mol Med Reports* 24:636,2021.<br>**Insult Treated:** Cardiac Hypertrophy<br>**Dosage:** 4-5 mg/kg/day; 7 treatments over 1-3 weeks
**MG149**<br>**AA analog**	Mof (Kat8; 47 μM[2])<br>Tip60 (74 μM[2])<br>p300 (>200 μM[4])<br>PCAF (>>200 μM[4])	**Citations:** *Circulation* 138:2820,2018.<br>**Insult Treated:** Ischemia-Reperfusion Injury<br>**Dosage:** 1 mg/kg/day for 14 days
**C646**	p300 (0.4 μM[2])<br>Tip60<br>CBP	**Citations:** *J Cell Mol Med* 23:3026,2019; *J Am Heart Assoc* 9:e017176,2020.<br>**Insults Treated:** Ischemia-Reperfusion Injury; Cardiac Hypertrophy<br>**Dosage:** 1 mg/kg/day for 14 days; 20 mg/kg/day 7 treatments over 3 weeks
**Curcumin**<br>**turmeric yellow**	p300 (25 μM[2]; >40 μM[1])<br>Tip60 (>200 μM[1])<br>CBP<br>PCAF	**Citations:** *J Clin Invest* 118:868,2008; *Cardiovas. Toxicol* 11:357,2011; *Free Radic Biol Med* 65:667,2013; *J Pharmacol Sci* 126:329,2014; *Drug Des Devel Ther* 10:1267,2016; *J Pharmacol Sci* 136:212,2018; *Biotechnol Adv (Review)* 38:107343,2020.<br>**Insults treated:** Cardiac Hypertrophy; Ischemia-Reperfusion Injury; Myocardial Infarction<br>**Dosage:** 5-200 mg/kg/day over 7-42 days (mostly via gavage)
**Garcinol**	PCAF (5 μM[5])<br>p300 (7 μM[5])<br>Tip60 (μMolar range[3])	**Citations:** *J Biol Chem* 279:33716,2004; AMB Express 10:137,2020.<br>**Insults Treated:** Cardiac Hypertrophy<br>**Dosage:** 10-100 mg/kg/day for 30 days (gavage)
**Pentamidine**	Tip60	**Citations:** *Mol Cancer* 9:34,2010; *J Neuroinflammation* 9:277.2012.<br>**Insults Treated:** none re cardiovascular disease; for colitis<br>**Dosage:** 0.8 – 4 mg/kg/day for 5 days
**NU9056**	Tip60 (2 μM[3])	**Citations:** *PLoS One* 7:e45539,2012; *eNeuro* 5:e0378-18,2018; *Frontiers Nutrition* 8:1,2021<br>**Insults Treated:** none re cardiovascular disease<br>**Dosage:** 2 -10 mg/kg/day for 1 day
**TH1834**	Tip60	**Citations:** *Biochem Soc Trans* 44:979,2016; *Omics* 23:457,2019; *Omics* 24:581,2020<br>**Insults Treated:** none re cardiovascular disease<br>**Dosage:** local only; none systemic
**Footnotes**	1. *Bioorg Med Chem* 17:1381;2009<br>2. www.selleckchem.com<br>3. *Biochem Soc Trans* 44:979;2016<br>4. www.medchemexpress.com<br>5. *J Biol Chem* 279:33716;2004	

Table S2. Cardiac Function in Infarcted TH1834-Treated Adult Mice Measured by M-Mode and Doppler Echocardiography. Ten week-old WT mice were subjected to MI surgery and injected with TH1834 (10mg/kg) or PBS on 14 consecutive days beginning three days after induction of MI. Data are mean ±SEM. LVAW d/s: left ventricle anterior wall thickness in diastole/systole; LVID d/s: left ventricular end diastolic/systolic diameter; LVPW d/s: left ventricle posterior wall thickness in diastole/systole; HR: heart rate; FS: fractional shortening; EF: ejection fraction; MPI: myocardial performance index; LV Vol d/s: left ventricular end-diastolic/systolic volume. *P<0.05 vs. Vehicle and †P<0.05 vs baseline value analyzed by two-way repeated measures ANOVA followed by Bonferroni's (effect of genotype) and Dunnett's (effect of time) multiple comparisons.

	Baseline		3 days post-MI		10 days post-MI		15 days post-MI		21 days post-MI		28 days post-MI	
**M-Mode & Doppler**	Vehicle N=12	TH1834 N=14	Vehicle N=12	TH1834 N=14	Vehicle N=12	TH1834 N=14	Vehicle N=12	TH1834 N=14	Vehicle N=12	TH1834 N=14	Vehicle N=12	TH1834 N=14
LVAW; d (mm)	0.605±0.029	0.645±0.027	0.879±0.055†	0.929±0.048†	0.830±0.037†	0.831±0.030†	0.824±0.024†	0.825±0.020†	0.833±0.034†	0.805±0.027†	0.768±0.035†	0.777±0.032†
LVAW; s (mm)	0.791±0.031	0.847±0.038	1.031±0.058†	1.086±0.033†	1.012±0.049†	1.061±0.033†	0.977±0.036†	1.062±0.027†	0.989±0.032†	1.025±0.035†	0.931±0.038†	0.990±0.040†
LVID; d (mm)	4.319±0.045	4.287±0.036	3.993±0.098†	4.056±0.076	4.730±0.146	4.521±0.113	4.910±0.197	4.585±0.124	5.007±0.210†	4.818±0.130†	5.047±0.150†	4.871±0.176†
LVID; s (mm)	3.397±0.050	3.353±0.046	3.279±0.110	3.334±0.082	3.982±0.144†	3.662±0.111	4.182±0.228†	3.671±0.130	4.253±0.238†	3.943±0.147†	4.300±0.171†	4.005±0.193†
LVPW; d (mm)	0.609±0.024	0.641±0.013	0.806±0.050†	0.709±0.040	0.712±0.062	0.779±0.041†	0.723±0.035	0.740±0.042	0.745±0.038†	0.769±0.042†	0.745±0.040†	0.754±0.054
LVPW; s (mm)	0.850±0.021	0.884±0.019	0.976±0.073	0.913±0.043	0.867±0.076	0.988±0.057	0.886±0.045	0.914±0.040	0.875±0.049	0.948±0.045	0.946±0.032	0.940±0.058
HR (bpm)	387.3±6.2	386.2±9.6	442.5±11.2†	410.1±17.5	445.0±16.2	441.3±16.4†	440.0±17.5†	423.7±15.4	433.0±16.0	434.5±18.9	430.6±19.9	444.4±14.9†
FS (%)	21.34±0.68	21.81±0.57	18.01±1.16	17.93±0.62†	15.95±0.69†	19.12±0.77*	15.31±1.25†	20.16±0.81*	15.50±1.22†	18.44±0.92	15.04±1.07†	18.25±1.11
MPI	0.424±0.003	0.432±0.006	0.523±0.004†	0.541±0.009†	0.545±0.010†	0.521±0.010†	0.570±0.012†	0.514±0.010†*	0.566±0.011†	0.520±0.009†*	0.568±0.011†	0.521±0.011†*
LV mass (mg)	75.36±4.16	79.75±3.11	101.7±7.6†	99.50±4.80†	119.8±8.9†	119.0±9.2†	127.6±7.5†	117.2±8.9†	137.5±11.5†	128.6±10.2†	131.3±10.2†	130.4±17.0†

[illegible]

Disease Models & Mechanisms • Supplementary information

Table S3. Cardiac Function in Infarcted TH1834-Treated Adult Mice Measured by B-Mode Echocardiography. Ten week-old WT mice were subjected to MI surgery and injected with TH1834 (10mg/kg) or PBS on 14 consecutive days beginning three days after induction of MI. Data are mean ± SEM. Area d/s: left ventricular end-diastolic/systolic area; FAC: fractional area change; FS: fractional shortening; EF: ejection fraction; SV: stroke volume; CO: cardiac output; LV Vol d/s: left ventricular end-diastolic/systolic volume. *P<0.05 vs. Vehicle and †P<0.05 vs baseline value analyzed by two-way repeated measures ANOVA followed by Bonferroni's (effect of genotype) and Dunnett's (effect of time) multiple comparisons.

	Baseline		3 days post-MI		10 days post-MI		15 days post-MI		21 days post-MI		28 days post-MI	
**B-Mode**	Vehicle N=12	TH1834 N=14	Vehicle N=12	TH1834 N=14	Vehicle N=12	TH1834 N=14	Vehicle N=12	TH1834 N=14	Vehicle N=12	TH1834 N=14	Vehicle N=12	TH1834 N=14
Area; d (mm^2)	24.38±0.51	24.96±0.53	26.07±0.87	26.55±0.76	33.71±2.40†	31.91±1.28†	36.78±2.48†	31.95±1.13†	36.16±1.96†	35.05±2.19†	37.69±1.72†	36.37±2.49†
Area; s (mm^2)	16.79±0.48	17.18±0.39	21.47±0.86†	21.60±0.73†	29.77±2.35†	25.81±1.23†	32.53±2.56†	25.85±1.21†	32.05±1.97†	29.65±2.14†	33.46±1.76†	30.92±2.53†
FAC (%)	31.24±0.77	31.16±0.65	17.76±1.42†	18.75±1.02†	12.13±0.99†	19.41±1.04†*	12.22±1.19†	19.45±1.19†*	11.74±0.89†	16.03±0.98†*	11.46±0.88†	15.91±1.23†*
EF (%)	43.60±1.02	44.16±0.78	26.38±1.92†	28.12±1.53†	18.54±2.40†	27.37±1.87†*	16.70±1.87†	28.53±1.70†*	17.13±1.74†	23.21±1.95†	16.91±1.65†	23.51±2.09†
SV (µL)	29.43±0.86	30.80±0.87	19.95±1.43†	21.96±1.33†	20.64±1.93†	28.10±1.33*	20.65±1.36†	29.95±1.19*	21.36±1.46†	27.06±1.35	22.66±1.19†	28.36±1.12*
CO (mL/min)	11.59±0.30	12.06±0.44	8.99±0.612†	9.20±0.56†	9.62±1.00	12.25±0.68	10.14±0.82	12.68±0.53	11.62±1.14	12.06±0.95	11.17±1.25	12.66±0.65
LV Vol; d (µL)	67.85±2.29	69.89±1.95	76.76±4.11	78.47±3.17	124.2±14.3†	107.7±7.8†	140.5±15.9†	109.5±7.0†	135.4±12.5†	128.7±14.0†	143.5±11.6†	136.9±15.8†
LV Vol; s (µL)	38.42±1.71	39.08±1.39	56.81±3.76†	56.51±2.71†	103.6±14.3†	79.65±7.39†	119.9±16.1†	79.59±6.72†	114.0±12.5†	101.6±13.2†	120.9±11.8†	108.6±15.3†

Table S4. Echocardiographic Function in Infarcted TH1834-Treated Adult Mice Calculated by Simpson's Method. Ten week-old WT mice were subjected to MI surgery and injected with TH1834 (10mg/kg) or PBS on 14 consecutive days beginning three days later. Data are mean ±SEM. FAC: fractional area change; FS: fractional shortening; EF: ejection fraction; SV: stroke volume; CO: cardiac output; LV Vol d/s: left ventricular end-diastolic/systolic volume. *P<0.05 vs. Vehicle and †P<0.05 vs baseline value analyzed by two-way repeated measures ANOVA followed by Bonferroni's (effect of genotype) and Dunnett's (effect of time) multiple comparisons.

	Baseline		3 days post-MI		10 days post-MI		15 days post-MI		21 days post-MI		28 days post-MI	
**Simpson's**	Vehicle N=8	TH1834 N=9	Vehicle N=8	TH1834 N=9	Vehicle N=8	TH1834 N=9	Vehicle N=8	TH1834 N=9	Vehicle N=8	TH1834 N=9	Vehicle N=8	TH1834 N=9
EF (%)	45.75±1.85	45.90±0.93	30.52±1.69†	29.53±1.49†	21.59±2.53†	31.86±2.34†	21.68±2.43†	32.54±2.56†*	21.03±2.63†	32.01±2.48†	19.55±2.50†	28.79±2.68†
FAC (%)	35.70±2.22	34.26±1.54	24.11±3.05	21.40±1.64†	15.58±3.10†	28.18±2.79	19.18±2.99†	28.99±2.67	15.00±4.17†	27.46±2.07†	11.10±3.10†	24.13±3.52†
SV (µL)	42.44±1.79	45.07±1.39	32.88±1.95†	29.83±1.24†	34.07±2.09	44.53±3.05†	38.46±2.53	46.90±3.11†	36.67±1.52	50.24±2.87†*	35.98±2.12	49.84±2.26†*
CO (mL/min)	17.08±0.67	17.94±0.61	15.05±0.89†	12.90±0.82†	14.96±0.99	18.66±1.30†	17.88±1.69	19.71±1.39†	20.36±3.18	22.46±1.69†	16.58±1.54	22.28±1.22†
LV Vol; d (µL)	93.25±3.82	98.33±2.95	108.9±6.8†	102.1±4.7	169.1±17.3†	143.2±9.5†	193.3±24.0†	148.8±10.2†	192.4±23.5†	168.2±19.3†	198.9±19.0†	187.8±22.1†
LV Vol; s (µL)	50.80±3.10	53.25±2.03	76.10±5.77†	72.35±4.47†	135.0±17.2†	98.75±9.28†	154.9±22.8†	101.9±10.0†	155.7±23.0†	117.9±18.4†	163.0±19.9†	138.0±21.2†

Table S5. Heart Weight/Body Weight Ratio 28 Days after MI or Sham. Ten-week-old WT mice were subjected to MI or sham surgery and injected with TH1834 (10 mg/kg) or PBS on 14 consecutive days beginning three days after surgery. Data are mean ± SEM.

	**Vehicle - Sham** N=5	**TH1834 - Sham** N=5	**Vehicle - MI** N=12	**TH1834 - MI** N=14
Body Weight	24.77±0.79	24.14±1.11	26.63±0.49	26.26±0.36
Tibia Length	16.66±0.09	16.7±0.1	16.84±0.05	16.88±0.05
Heart Weight	104.42±3.92	109.24±5.02	131.9±3.9	126.0±2.7
Heart Weight/Body Weight	4.21±0.06	4.55±0.23	4.95±0.10	4.81±0.10
Heart Weight/Tibia Length	6.26±0.2	6.54±0.29	7.83±0.23	7.47±0.17
Wet Lung/Dry Lung	4.1±0.04	4.08±0.04	4.03±0.04	4.00±0.05

Table S6. Antibodies for Immunofluorescent Staining.

Antigen	Manufacturer	Catalog #	Made in	Dilution
1° 5'-bromodeoxyuridine (BrdU)	Abcam	ab6326	rat	1:200
2° goat anti-rat 594	Invitrogen	A-11007	goat	1:500
1° caspase-3	Cell Signaling	9661S	rabbit	1:50
2° goat anti-rabbit 594	Invitrogen	A-11037	goat	1:500
1° connexin-43	Abcam	ab11370	rabbit	1:1000
2° goat anti-rabbit 488	Invitrogen	A-11034	goat	1:500
1° phospho-histone H3 (pH3)	EMD Millipore	06-570	rabbit	1:400
2° goat anti-rabbit 594	Invitrogen	A-11037	goat	1:500
1° Ki67	Invitrogen	14-5698-82	rat	1:250
2° goat anti-rat 594	Invitrogen	A-11007	goat	1:500
1° phosphorylated Atm (pAtm)	Novus	NB100-306	mouse	1:200
2° goat anti-mouse 488	Invitrogen	A-11029	goat	1:500
2° goat anti-mouse 568	Invitrogen	A-11031	goat	1:500
1° cardiac-Troponin-T (cTnT)	Abcam	ab8295	mouse	1:200
2° goat anti-mouse 488	Invitrogen	A-11029	goat	1:500
1° α-actinin	Abcam	ab68167	rabbit	1:500
2° goat anti-rabbit 494	Invitrogen	A-11037	goat	1:500
2° goat anti-rabbit 488	Invitrogen	A-11034	goat	1:500

Table S7. Primers/Probes for Taqman qRT-PCR Gene Expression Analysis

**Gene Target**	**Taqman Probe Kit** (Thermo-Fisher catalog #)
Bax	Mm00432051_m1
Bbc3 (Puma)	Mm00519268_m1
Cdkn1a (p21)	Mm00432448_m1
Cdkn1b (p27)	Mm00438168_m1
Gapdh	Mm99999915_g1
Meis1	Mm00487664_m1
Rpl37a	Mm01546394_s1
TP53 (p53)	Mm01731290_g1
Vcam1	Mm01320970_m1
Wee1	Mm00494175_m1
